# A Randomized Controlled Trial of Three Advanced Wound Dressings in Split-Thickness Skin Grafting Donor Sites—A Personalized Approach?

**DOI:** 10.3390/jpm12091395

**Published:** 2022-08-27

**Authors:** Andrzej Hecker, David Benjamin Lumenta, Petra Brinskelle, Isabelle Sawetz, Andreas Steiner, Birgit Michelitsch, Herwig Friedl, Daniel Gmainer, Lars-Peter Kamolz, Raimund Winter

**Affiliations:** 1 Division of Plastic, Aesthetic and Reconstructive Surgery, Department of Surgery, Medical University of Graz, Auenbruggerplatz 5, 8036 Graz, Austria; 2 Research Unit for Digital Surgery, Division of Plastic, Aesthetic and Reconstructive Surgery, Department of Surgery, Medical University of Graz, Auenbruggerplatz 5, 8036 Graz, Austria; 3 Institute of Statistics, Graz University of Technology, Kopernikusgasse 24/III, 8010 Graz, Austria

**Keywords:** split-thickness skin graft donor sites, wound dressing, personalized medicine, nanocellulose, wound management, pain relief

## Abstract

Background: Split-thickness skin grafting (STSG) is a frequently used reconstructive technique, and its donor site represents a standardized clinical model to evaluate wound dressings. We compared hydroactive nanocellulose-based, silver-impregnated and ibuprofen-containing foam wound dressings. Methods: A total of 46 patients scheduled for elective surgery were evaluated on the STSG donor site for wound healing (time-to-healing, Hollander Wound Evaluation Scale), pain level (Visual Analogue Scale), and handling (ease of use), as well as scar quality (Patient Scar Assessment Scale, Vancouver Scar Scale) after 3, 6 and 12 months. Results: Almost all dressings compared equally well. We observed statistically relevant differences for pain level favoring the ibuprofen-containing dressing (*p* = 0.002, ΔAIC = 8.1), and user friendliness in favor of nanocellulose (dressing removal: *p* = 0.037, ΔAIC = 2.59; application on patient: *p* = 0.042, ΔAIC = 2.33; wound adhesion: *p* = 0.017, ΔAIC = 4.16; sensation on skin: *p* = 0.027, ΔAIC = 3.21). We did not observe any differences for wound healing across all groups. Treatment with hydroactive nanocellulose and the ibuprofen-containing foam revealed statistically relevant better scar appearances as compared to the silver wound dressing (*p* < 0.001, ΔAIC = 14.77). Conclusion: All wound dressings performed equally well, with the detected statistical differences hinting future directions of clinical relevance. These include the reserved use of silver containing dressings for contaminated or close to contaminated wounds, and the facilitated clinical application of the nanocellulose dressing, which was the only suitable candidate in this series to be impregnated with a range of additional therapeutic agents (e.g., disinfectants and pain-modulating drugs). Personalized donor site management with the tested dressings can meet individual clinical requirements after STSG and improve management strategies and ultimately patient outcomes.

## 1. Introduction

Split-thickness skin grafting (STSG) is a frequently used reconstructive technique to close skin defects. Harvesting split-thickness skin creates a new partial thickness wound on the chosen donor site [1]. Among the various wound dressings for STSG donor site management [2,3,4], an ideal one should promote rapid wound healing, reduce pain, especially during dressing changes, decrease scar formation and be easy to handle [1,5,6]. The available dressing types, the required clinical application and individual patient requirements decide upon the appropriate choice amongst them [2,7,8,9,10]. Antimicrobial effects of silver-impregnated dressings as well as pain-relieving effects of ibuprofen-containing dressing have proven beneficial previously [11,12].

The ubiquitous “fat-impregnated gauze” for STSG donor sites, which is allowed to dry and is left on the wound until spontaneous removal is possible, can be regarded as a relatively inexpensive standard dressing. Newer wound dressings can provide a protective (epithelial-like) wound environment, which aids in accelerating epithelization and promoting less pain during dressing changes [2,13]. The recent addition of bacterial nanocellulose (epicite + hydro, QRSKIN GmbH, Würzburg, Germany) to this type of advanced dressing has demonstrated promising results in partial-thickness burn wounds [14]. Nanocellulose can be impregnated with locally active disinfectants or anti-inflammatory agents [15] and is customizable for different clinical indications [16,17,18,19]. The current trend is to replace traditional dressings with technologically more advanced and individualized dressings, providing a more personalized management [20].

In this study, we compared three advanced synthetic wound dressings with different properties in a standardized fashion. In contrast to the silver-impregnated foam [11] and ibuprofen-containing foam [12,21,22], there is scant clinical evidence about the recent nanocellulose-based dressing [14], and this is the first prospective randomized clinical trial comparing the three advanced dressings in STSG donor sites. The purpose of this study was to evaluate their healing properties (days until complete re-epithelialization, standardized wound evaluation), dressing-related pain (pain intensity before, during and after dressing changes and at rest/motion), and ease of handling (evaluation by healthcare personnel and patient).

## 2. Materials and Methods

### 2.1. Study Design

This study was designed as a monocentric, open, prospective, randomized, controlled clinical trial and was conducted at the Medical University of Graz, Austria (Division of Plastic, Aesthetic and Reconstructive Surgery, Department of Surgery) from May 2016 to April 2020. Advanced wound dressings were applied directly in the operating room, and parameters (healing properties, dressing-related pain, dressing handling, scar quality) were evaluated in the inpatient setting, and in the outpatient setting after discharge from the hospital. The study design and protocol were approved by the institutional ethical review board (Ethical Board Approval No.: 28-405 ex 15/16). This study is registered in “Deutsches Register Klinischer Studien” (ID: DRKS00030018). Written informed consent was obtained from the participants prior to study enrolment.

### 2.2. Patient Selection

Female and male patients scheduled for elective skin grafting were eligible for inclusion in the study. Exclusion criteria were age under 18 years, pregnancy/lactation, local infection at planned STSG donor site, hypersensitivity to dressing components, malignant or autoimmune disease, vasculitis, connective tissue diseases, immune system disorders, chemo-, radio- and immunosuppressive therapy within 30 days prior to study enrolment, systemic corticosteroids (intake > 10 mg/day), drug abuse, excessive alcohol consumption, recent participation in other clinical trials within 4 weeks before study enrolment.

### 2.3. Wound Dressing Material

The ibuprofen-containing foam, Biatain^®^ Ibu (Coloplast A/S, Humlebæk, Denmark), is a non-adhesive foam dressing which is made of hydrophilic polyurethane hydrocellular. Biatain^®^ Ibu contains incorporated ibuprofen (0.5 mg/cm^2^), a non-steroidal anti-inflammatory drug. In the presence of wound fluid this foam topically releases its incorporated ibuprofen [12].

The silver-impregnated foam, Mepilex^®^ Ag (Mölnlycke, Göteborg, Sweden) is an antimicrobial foam dressing which consists of a soft silicone wound contact layer and an absorbent polyurethane foam layer. The polyurethane foam layer contains activated carbon and silver (silver concentatrion 1.2 mg/cm^2^) [11].

The nanocellulose, epicite + hydro (QRSKIN GmbH, Würzburg, Germany), is a semi-transparent biomaterial made of bacterial nanocellulose synthesized by Komagataeibacter xylinus and which consists of 95% water [14].

### 2.4. Treatment Allocation and Schedule

Eligible patients were randomized to treatment with either of the three dressings. Randomization was conducted in a 1:1:1 ratio. The randomization list that was generated was prepared in advance in the order of the study preparation. The allocation codes were sent to attending surgeons on the day of the operation prior to elective STSG. All skin grafts were taken from the anterior thigh area in a standardized fashion by use of an electric dermatome with a common cut depth of 0.4 mm. The donor area was measured (length multiplied by width). After applying the assigned wound dressing material, the secondary dressings consisted of a fat-containing gauze (Grassolind^®^, Hartmann, Vienna, Austria) only for the nanocellulose, and then for all dressings of sterile dry gauze (Gazin^®^, Lohmann & Rauscher Intl., Rengsdorf, Germany) and adhesive bandage (Cosmopor^®^ E, Hartmann, Vienna, Austria). Pain at rest and in motion was evaluated daily until the 14th postoperative day using a 10-point visual analogue scale (VAS). Additional pain scores were obtained before, during and after the dressing change on the 14th postoperative day. Usability of the dressing material was evaluated by means of a patient- and healthcare personnel-related self-provided 4-item Likert scale questionnaire. After a follow-up period of 3, 6 and 12 months, all donor sites were evaluated using the Vancouver Scar Scale (VSS) [23] and the Patient Scar Assessment Scale (PSAS) [24].

### 2.5. Treatment Regime of Wound Dressings

All initial wound dressings and changes were performed by plastic surgeons. After harvesting the split-thickness skin graft with an electric dermatome (depth 0.4 mm) from the donor site, the primary wound dressing (Biatain^®^ Ibu, Coloplast A/S, Humlebæk, Denmark; Mepilex^®^ Ag, Mölnlycke, Göteborg, Sweden; epicite + hydro, QRSKIN GmbH, Würzburg, Germany) was applied directly to the superficial wound during surgery in the operating room.

For the epicite + hydro (QRSKIN GmbH, Würzburg, Germany) group, a moisture-protecting layer (Grassolind^®^, Hartmann, Vienna, Austria) was put in between the primary wound dressing and the sterile dry gauze (Gazin^®^, Lohmann & Rauscher Intl., Rengsdorf, Germany) in accordance with the manufacturer’s instructions before fixation with an adhesive bandage (Cosmopor^®^ E, Hartmann, Vienna, Austria).

For Biatain^®^ Ibu (Coloplast A/S, Humlebæk, Denmark) and Mepilex^®^ Ag (Mölnlycke, Göteborg, Sweden), the sterile gauze was directly applied to the primary dressing before fixation with an adhesive bandage (Cosmopor^®^ E, Hartmann, Vienna, Austria).

Until complete re-epithelialization, the primary wound dressings (Biatain^®^ Ibu, Mepilex^®^ Ag) were left in place. In the case of epicite + hydro, the fat-containing gauze on top of the primary dressing was also left in place. In all groups, the secondary wound dressings (sterile dry gauze, adhesive bandage) were only changed when necessary (e.g., exuding wound).

The dressing regime is structured as follows, starting from the bottom (direct wound contact):

(1) Primary wound dressing: Biatain^®^ Ibu (Coloplast A/S, Humlebæk, Denmark), Mepilex^®^ Ag (Mölnlycke, Göteborg, Sweden), epicite + hydro (QRSKIN GmbH, Würzburg, Germany)
(2) Secondary wound dressing (only for epicite + hydro): fatty gauze;
(3) Secondary wound dressing: sterile dry gauze;
(4) Secondary wound dressing: adhesive bandage.

### 2.6. Study Assessments and Endpoints

The primary outcome was time-to-healing (days until complete re-epithelialization); wound assessment following dressing removal using the validated Hollander Wound Evaluation Scale (HWES) [25]; intensity of pain before, during and after dressing change and at rest/motion using VAS (0 indicating no pain; 10 indicating unbearable pain); extent of subjective dressing handling (ease of use) after dressing changes using a 4-item Likert scale questionnaire (0 indicating favorable/very good/very satisfied; 3 indicating painful/bad/unsatisfactory) for patients (sensation on skin after application, sensation on skin during dressing change, redness/tolerance, discoloration) and for healthcare personnel (removal of dressing, abnormality after dressing removal, removal from wound, application on patient, pain by patient, smelling, usability of dressing material, application on wound, wound adhesion). The secondary outcome was scar quality assessment using the VSS and the PSAS (1 = normal skin to 10 = worst scar imaginable) at 3, 6 and 12 months following STSG donor site collection. The VSS was performed by a blinded rater (plastic surgeon) and calculated by the cumulative scoring system (pigmentation: 0 = normal to 3 hyperpigmented; height: 0 = flat to 3 =>5 mm; pliability: 0 = normal to 5 = contracture; vascularity: 0 = normal to 3 = purple; minimum 0 to maximum 14 points). Re-epithelialization, HWES, pain scoring (by asking the patient for current VAS score), dressing handling questionnaire and VSS were done by involved study doctors (several different plastic surgeons). Subjective dressing handling and PSAS, however, were evaluated by the included study patients themselves.

### 2.7. Statistical Analysis

Data were analyzed with the statistical software R (version 4.1.2; R Core Team, 2021) [26]. The statistical evaluation included means or medians and standard deviations or interquartile ranges or ranges of continuous or ordered variables, and relative frequencies of categorical factors. Ordered logistic regression, especially proportional odds log-log models [27] with a stepwise elimination of irrelevant factors were used for statistical analyses. The size of a significant effect in a model is estimated through the difference in the Akaike Information Criteria (ΔAIC) when omitting such a variable from the model. The intention-to-treat principle was applied. All reported *p*-values corresponded to tests on two-sided alternatives and were considered statistically significant when at most *p* < 0.05.

## 3. Results

**Figure 1:**
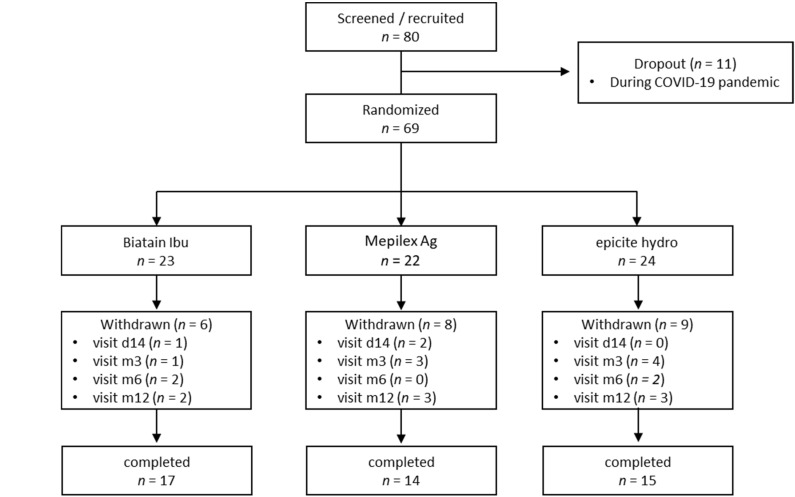
Flow of patients through the trial. d14, 14 days after dressing application; m3/m6/m12, 3/6/12 months following STSG donor site collection.

From 2016 until 2020 a total of 69 eligible patients (80 recruited, 11 dropouts) were randomized to nanocellulose (*n* = 24), silver-impregnated foam (*n* = 22), or ibuprofen-containing foam (*n* = 23). Among the 69 patients the main reason for operation were skin lesions (29%), burns (28%), trauma (22%), pressure ulcers (13%) and other (8%). Demographic variables and baseline STSG donor site characteristics were comparable (no significant differences) across all groups (Table 1). Among the 69 patients, 23 patients withdrew prior to study completion. After inpatient discharge, these 23 patients did not attend outpatient follow-up. Visit-related premature withdrawals are shown in Figure 1. In total, 46 patients completed the study (nanocellulose: 15; silver-impregnated foam: 14; ibuprofen-containing foam: 17). The requirement for a dressing change before the 14th postoperative day was in decreasing order 93% (14/15), 64% (9/14) and 41% (7/17) for nanocellulose, silver-impregnated foam, and ibuprofen-containing foam, respectively. A total of 66 patients received a regular non-opioid pain medication with metamizol; in case of a metamizol allergy, diclofenac with orphenadrincitrat was used. Only three patients (one in each group) required additional pain-relieving medication for the STSG donor site.

### 3.1. Wound Healing

The median time-to-healing did not differ in a statistically significant manner among the three groups: nanocellulose (14 days; range: 12–36), silver-impregnated foam (16 days; range: 11–22) and ibuprofen-containing foam (16 days; range: 11–21). The same applied to the HWES, which revealed no statistical differences between the three groups.

### 3.2. Pain

**Figure 2:**
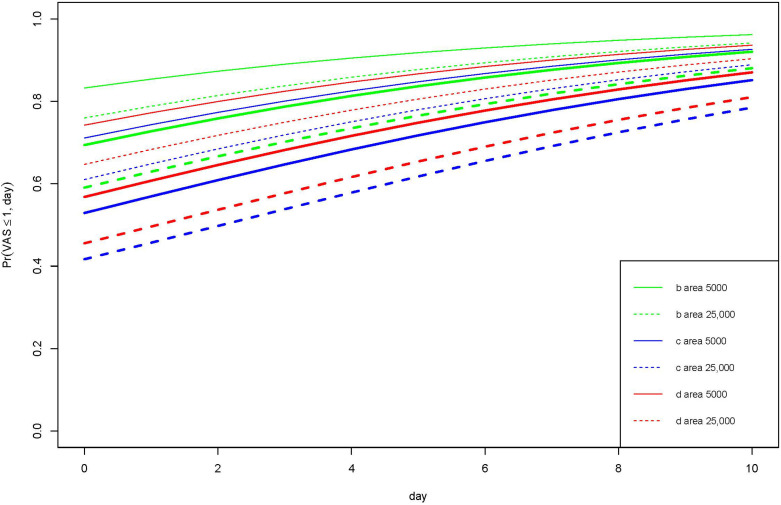
Probability for experiencing almost no pain (VAS score ≤ 1) at rest (thin lines) or in motion (thick lines) with ibuprofen-containing foam (b, green), silver-impregnated foam (c, blue) and bacterial nanocellulose (d, red). Small wounds (wound area 5000 mm^2^; solid line) had a higher probability of experiencing less pain compared to larger wounds (wound area 25,000 mm^2^; dotted line). Pr, probability; VAS, visual analogue score.

Average pain levels were highest during (nanocellulose: 1.2, standard deviation (SD) ± 2.7; silver-impregnated foam: 1.0, SD ± 2.0; ibuprofen-containing foam: 1.6, SD ± 2.7) followed by after (nanocellulose: 0.2, SD ± 0.5; silver-impregnated foam: 0.4, SD ± 1.2; ibuprofen-containing foam: 0.7, SD ± 1.2) and before (nanocellulose: 0.2, SD ± 0.6; silver-impregnated foam: 0.4, SD ± 0.7; ibuprofen-containing foam: 0.1, SD ± 0.4) a dressing change. The average VAS scores of the dressing-change-related pain scores were low across all groups (ibuprofen-containing foam 0.8 (SD ± 1.9), silver-impregnated foam 0.6 (SD ± 1.4), nanocellulose 0.5 (SD ± 1.7)). The probability for experiencing almost no pain (VAS score ≤ 1) at rest (thin lines in Figure 2) or in motion (thick lines in Figure 2) was highest in the ibuprofen-containing foam group followed by nanocellulose and silver-impregnated foam (*p* = 0.002, ΔAIC = 8.1, Table 2). The probability to experience almost no pain at rest as compared to in motion was statistically similar in all groups (*p* < 0.001, ΔAIC = 24.9, Table 2). Furthermore, smaller wounds (wound area 5000 mm^2^ vs. 25,000 mm^2^) had a higher probability of experiencing no pain (*p* < 0.001, ΔAIC = 25.8, Table 2). All STSG donor sites became less painful over the observation period of 14 days (*p* < 0.001, ΔAIC = 31.5). In addition, patients with smaller ASA (American Society of Anesthesiologists) risk classification experienced less pain (*p* < 0.001, ΔAIC = 23.4).

### 3.3. Handling of Dressings

#### 3.3.1. Subjective Dressing Handling Evaluation by Patient

Patient experiences related to skin sensation after dressing application was statistically superior in nanocellulose (23/24, 95.8%) as compared to silver-impregnated foam (20/22, 90.9%) and ibuprofen-containing foam (16/23, 69.6%) (*p* = 0.027, ΔAIC = 3.21). Sensation on skin during dressing changes, redness/tolerance and discoloration revealed no statistically differences among all groups.

#### 3.3.2. Subjective Dressing Handling Evaluation by Healthcare Personnel

From the healthcare personnel’s perspective, dressing removal from the wound (*p* = 0.037, ΔAIC = 2.59) and application on the wound (*p* = 0.042, ΔAIC = 2.33) was significantly better with nanocellulose (21/24, 87.5%) compared to silver-impregnated foam (19/22, 86.5%) followed by ibuprofen-containing foam (14/23, 60.9%). Significantly less wound adhesion was observed in nanocellulose (22/24, 91.7%) compared to ibuprofen-containing foam (14/23, 60.9%) followed by silver-impregnated foam (13/22, 59.1%) (*p* = 0.017, ΔAIC = 4.16).

### 3.4. Scar Evaluation

#### 3.4.1. PSAS by Patient

Scores for pain, itching, stiffness, thickness and irregularity after 3, 6 and 12 months were generally low across all groups. After 3 months, median wound color scores were in the mid-range (nanocellulose: 6, silver-impregnated foam: 7, ibuprofen-containing foam: 4) and decreased from 3 to 12 months after STSG donor site collection for all dressings (Table S1). The probability for experiencing no itching (PSAS score ≤ 1) was significantly higher with nanocellulose and ibuprofen-containing foam compared to silver-impregnated foam (*p* < 0.001, ΔAIC = 14.85). The probability for no color change was significantly higher with ibuprofen-containing foam followed by nanocellulose and silver-impregnated foam (*p* = 0.033, ΔAIC = 2.85, Table S1, Figure S1). Scar color improved in a time-dependent manner over 12 months (*p* < 0.001, ΔAIC = 43.37, Table S1, Figure S1). The probability for experiencing pain, stiffness, thickness and irregularity were not significantly different between dressing groups.

#### 3.4.2. VSS by Observer

Scores for vascularity, height, pigmentation and pliability after 3, 6 and 12 months were low across all groups (Table S1). The probability for normal pliability and height was significantly higher in nanocellulose and ibuprofen-containing foam compared to silver-impregnated foam (*p* = 0.0005, ΔAIC = 11.09 for pliability and *p* = 0.005, ΔAIC = 6.58 for height, Table S1). All VSS observations demonstrated a time-dependent manner over 12 months (*p* < 0.0001, ΔAIC = 23.35 for vascularity, *p* < 0.0001, ΔAIC = 14.77 for pigmentation, *p* = 0.0005, ΔAIC = 10.01 for height, and *p* = 0.033, ΔAIC = 2.54 for pliability, Table S1).

## 4. Discussion

In this study, all applied wound dressings, with routine off-the shelf availability at our institution, revealed comparable re-epithelialization properties and overall donor site outcome. Among the tested dressings, the ibuprofen-containing foam demonstrated a statistically superior pain relief at rest or in motion (but not during dressing changes), nanocellulose had the highest user-friendliness, and nanocellulose as well as the ibuprofen-containing foam demonstrated an improved scar quality in the long-term.

Time-to-healing (=completed re-epithelialization) and HWES were comparable across all groups. We found that the median time-to-healing was between 14 and 16 days for all groups [1]. In contrast, conventional management of STSG donor sites with fat-impregnated gauzes have a reportedly longer time-to-healing ranging from 16 to 19 days [28,29]. In general, moist dressing types have clinical advantages over non-moist dressings in the management of STSG donor sites [1,13]. This is in support of the study design, where we tested advanced (moist) dressings only, and this can explain the shorter time-to-healing in moist dressing environments as compared to traditionally applied fat-impregnated gauzes [2,13].

Pain at the donor site during the postoperative period was consistently low for all dressing materials. The mean pain assessed by the 10-point VAS score was less than 1.6 in all groups during dressing changes. Considering only a VAS score >3 as clinically relevant [30], our statistically significant findings were unlikely to be of clinical relevance, and any of the tested dressing materials were equally useful for alleviating pain on STSG donor sites. Of note, only one patient in each group required additional pain-relieving medication for the STSG donor site.

In all groups, the subjective handling and user friendliness either by patients (sensation on skin after application) or healthcare personnel (removal from wound, application on patient, wound adhesion) revealed a superior outcome for the nanocellulose-based dressing as compared to the other two dressings. Previous reports confirmed silver-impregnated foams to be easy to apply and a comfortable dressing, underlining the higher level of applicability of the dressings tested in this study [11]. In our study, the ibuprofen-containing foam scored by comparison less well during dressing removal and application, but demonstrated the best pain relief at rest or in motion. Despite containing ibuprofen, this observation most likely indicates the agent itself being effective for the duration of its active diffusion in the initial phase, but not for the dressing change on day 14. Dressings that can be easily removed have a positive effect on wound healing by decreasing pain levels during dressing changes [31,32,33]. The improved exudate management of the nanocellulose-based dressing and its molding capabilities make it clinically more useful in challenging anatomic regions prone to fine movement and skin wrinkling as compared to the foam-like structure of ibuprofen-containing foam or silver-impregnated foam (e.g. buttocks, back). Both foam dressings are more dependent on wound adherence, but tend to reposition themselves after intermittent dislocation.

Regarding scar quality (pliability, itching) and aesthetic appearance (color, height), the silver-impregnated foam showed a less favorable outcome compared to the other dressings. Silver-impregnated dressings were reported to cause discoloration resulting from occupational exposure or ingestion of colloidal silver rather than topical application [34]. Nevertheless, there seems to be an association between application of silver dressings and scar discoloration, especially when used in clean wounds [35]. Various studies have attributed a prophylactic antimicrobial effect of silver-impregnated foam to the bactericidal properties of ionic silver contained in its foam [36,37]. No infection was registered in any of our groups. In clean wounds like STSG donor sites, silver dressings demonstrated therefore no advantage and are only recommended for use in infected or near infected wounds [38]. The indications for the use of silver ion dressings on non-infected wounds remain controversial [39]. Whether the use of the used foam dressing without silver would have provided an equally satisfactory outcome in STSG donor sites as compared to the other two dressings remains elusive and was not evaluated in our study.

For none of the tested dressings was an adverse event reported, underlining the safety and high biocompatibility in STSG donor sites. Ibuprofen-containing foam was the only dressing with a pharmacologically active pain-relieving non-steroidal anti-inflammatory drug. Nanocellulose contained no pharmacologically active ingredients, and considering the fluid-soaking capability of nanocellulose it was the only dressing capable of being loaded with a range of different drugs or ingredients, making it a promising all-round carrier for various additives directed at individualized future wound care [16,17,18,19].

In our view, all tested dressings are suitable for the treatment of STSG donor sites. For a patient-specific personalized approach, our results hint at future directions of clinical relevance. The ibuprofen-containing foam could be of particular interest to pain-sensitive patients. Larger wound areas and higher ASA risk classification had a higher probability of experiencing pain. Thus, patients with larger wound areas and/or higher ASA risk classification could also benefit from the ibuprofen-containing foam ultimately leading to improved wound healing [32].

We recommend a reserved use of silver-impregnated foam for contaminated or infected wounds [36,37]. Patients who are more prone to infections, for example due to immunodeficiency or with ongoing immunosuppressive treatment, may benefit from the prophylactic antimicrobial effect of silver-impregnated foam.

Nanocellulose, as an all-round carrier for different additives, offers the possibility of a personalized approach by a single wound dressing. To reduce the risk of infection, the dressing can be impregnated with a disinfectant or locally active antibiotics [17]. Using the nanocellulose to monitor the wound pH, it is possible to identify a deteriorating wound environment at an early stage [18]. An increase in the pH of the wound dressing can be sign of incipient infection. [40] This type of customized wound treatment can trigger a timely switch to a suitable anti-infective therapy based on measured pH changes. The versatile features of nanocellulose make it a suitable candidate for an almost “ideal” wound dressing, which enables a personalized approach.

Personalized donor site management with the tested dressings can meet individual clinical requirements after STSG, improve management strategies, and ultimately patient outcomes.

## 5. Limitations

The final study endpoints were evaluated in 46 patients in this monocentric trial and all patients routinely received analgesics during the first three postoperative days irrespective of the pain experienced on the STSG donor sites. Evaluation of wounds and scars was based on clinical scoring questionnaires (HWES, VSS) associated with limited objectivity and limited to the experience of the investigating observers. All patients undergoing STSG are routinely advised to maintain adequate sun protection for STSG donor sites to minimize the effects of UV-related scar discoloration. Bias by individual factors on scar development (e.g., genetic predisposition, skin quality) and compliance to recommended postoperative regimes may also be considered as limiting factors.

## 6. Conclusions

All tested dressing materials demonstrated almost equally favorable outcomes and the detected statistical differences may be of minor clinical relevance. Most importantly, the results are useful for future directions of personalized approaches to STSG donor site management or clean wounds in general, and include: the pain-alleviating properties of the ibuprofen-containing foam, the reserved use of silver containing dressings for contaminated or in proximity of infected wounds, and the carrier potential of the nanocellulose dressing. Nanocellulose revealed in its “pure” form favorable properties by comparison to the other two “loaded” dressings and was the only tested “carrier” candidate suitable to be impregnated with a range of additional therapeutic agents (e.g., disinfectants, pain modulators).

A personalized approach in STSG donor sites management has the potential to meet the individual clinical needs of each patient, promote better therapy, and subsequently improve outcomes. The observed differences among the tested materials is based on the study’s small sample size, and results should therefore be interpreted with caution.

## Figures and Tables

**Table 1:** Demographic variables and baseline characteristics. ASA, American Society of Anesthesiologists; IR, interquartile range.

Total N = 69	Biatain^®^ Ibu (N = 23)	Mepilex^®^ Ag (N = 22)	Epicite + Hydro (N = 24)
Age (years), mean (SD)	48 ( ± 18)	54 (±20)	47 (±17)
Gender, *n* (%)			
Male	17 (73.9)	10 (45.5)	18 (75.0)
Female	6 (26.1)	12 (54.5)	6 (25.0)
BMI (kg/m^2^), median (IR)	26.0 (7.6)	25.4 (5.7)	27.1 (6.0)
Length of stay (days intervention—discharge), median (range)	8 (0–22)	8 (2–28)	9 (4–23)
Smoking, *n*	9	11	8
ASA risk classification			
ASA 1, *n*	5	8	11
ASA 2, *n*	14	4	9
ASA 3, *n*	4	10	4
ASA 4, *n*	0	0	0
Wound size (mm^2^), median (IR)	6800 (8400)	6800 (12,675)	10,650 (22,525)

**Table 2:** The probability for experiencing almost no pain (VAS score ≤ 1) at rest or in motion in a wound area of 5000 mm^2^ or 25,000 mm^2^. VAS, Visual Analogue Scale.

Probability for ExperiencingVAS Score ≤ 1	Ibuprofen-Containing Foam			Silver-Impregnated Foam			Nanocellulose		
	Day 0	Day 2	Day 4	Day 0	Day 2	Day 4	Day 0	Day 2	Day 4
Wound area 5000 mm^2^									
In rest, (%)	83	87	91	71	77	83	74	80	85
In motion, (%)	69	76	81	53	61	68	57	65	72
Wound area 25,000 mm^2^									
In rest, (%)	76	81	86	61	68	75	65	72	78
In motion, (%)	59	67	73	42	50	58	46	54	62

## Data Availability

Detailed data supporting the results are available at the authors.

## Supplementary Materials

The following supporting information can be downloaded at: https://www.mdpi.com/article/10.3390/jpm12091395/s1, Figure S1: Scar colour change of ibuprofen-containing foam, silver-impregnated foam and nanocellulose in a time-dependent-manner over 12 months; Table S1: PSAS scores surveyed by patients and VSS scores surveyed by observers after 3, 6 and 12 months.
