# Supplementary material for: A Randomized Controlled Trial of Three Advanced Wound Dressings in Split-Thickness Skin Grafting Donor Sites—A Personalized Approach?
Source: J Pers Med. 2022 Aug 27;12(9):1395. doi: 10.3390/jpm12091395 (PMC9506097; doi:10.3390/jpm12091395)
Supplement: Supplementary file 1 [file jpm-12-01395-s001.zip › jpm-1884098-supplementary.pdf]

Supplementary Material

Figures:

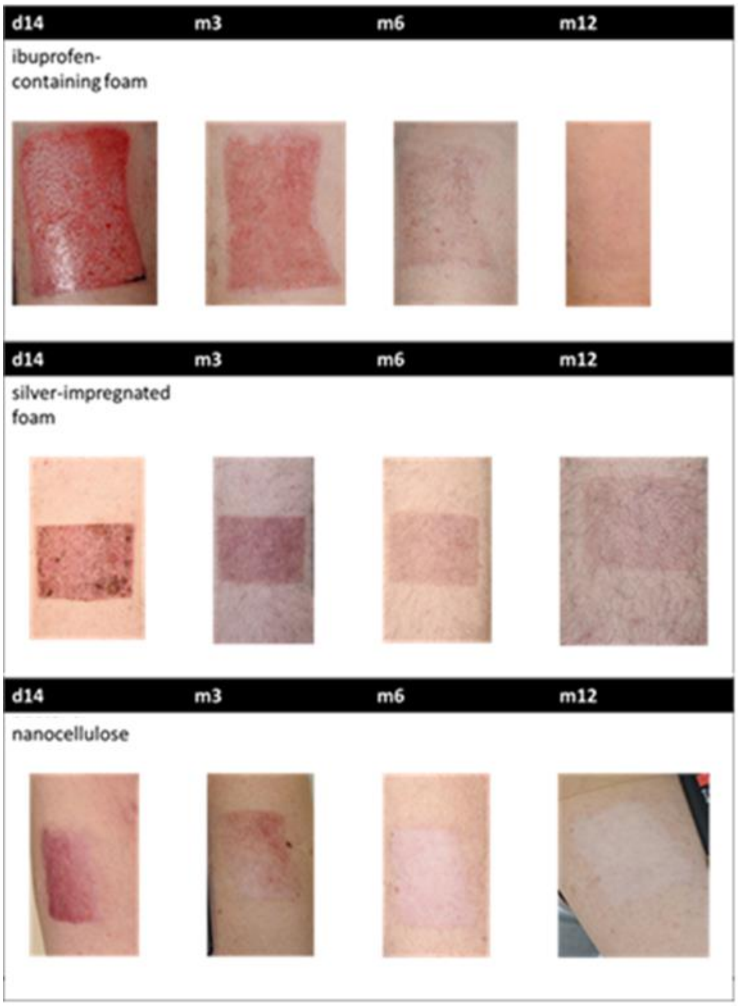

Figure S1. Scar colour change of ibuprofen-containing foam, silver-impregnated foam and nanocellulose in a time-dependent-manner over 12 months. d14, 14 days after dressing application; m3 / m6 / m12, 3 / 6 / 12 months following STSG donor site collection.

Tables:

Table S1. PSAS scores surveyed by patients and VSS scores surveyed by observers after 3, 6 and 12 months. PSAS, Patient Scar Assessment Scale; VSS, Vancouver Scar Scale; IR, interquartile range.

	nanocellulose	silver-impregnated foam	ibuprofen-containing foam
**PSAS patient, median (IR)**			
Pain 3 months	1 (0)	1 (0)	1 (1)
Pain 6 months	1 (0)	1 (0)	1 (0)
Pain 12 months	1 (0)	1 (0)	1 (0)
Itching 3 months	1 (0)	1 (4)	1 (0)

Itching 6 months	1 (0)	1 (1)	1 (0)
Itching 12 months	1 (0)	1 (1)	1 (0)
Colour 3 months	6 (2.5)	7 (3)	4 (5)
Colour 6 months	3 (1.75)	5 (3.5)	3.5 (3)
Colour 12 months	2 (2)	3 (4)	2(1)
Stiffness 3 months	1 (0)	1 (0)	1 (0)
Stiffness 6 months	1 (0)	1 (0)	1 (0)
Stiffness 12 months	1 (0)	1 (0)	1 (0)
Thickness 3 months	1 (0)	1 (1)	1 (0)
Thickness 6 months	1 (0)	1 (1)	1 (0)
Thickness12 months	1 (0)	1 (0)	1 (0)
Irregularity 3 months	1 (0)	1 (0)	1 (0)
Irregularity 6 months	1 (0)	1 (0)	1 (0)
Irregularity 12 months	1 (0)	1 (0)	1 (0)
**VSS observer, median (IR)**			
Vascularity 3 months	1 (3)	1 (2)	1 (1)
Vascularity 6 months	0 (1)	0 (1)	1 (1)
Vascularity 12 months	0 (1)	0 (1)	0 (0)
Pigmentation 3 months	2 (2)	2 (1)	2 (2)
Pigmentation 6 months	0 (1)	1 (2)	2 (2)
Pigmentation 12 months	0 (1)	0 (1)	0 (1)
Pliability 3 months	0 (1)	1 (2)	0 (0)
Pliability 6 months	0 (1)	0 (1)	0 (0)
Pliability 12 months	0 (1)	0 (1)	0 (0)
Height 3 months	0 (0)	0 (1)	0 (1)
Height 6 months	0 (0)	0 (1)	0 (0)
Height 12 months	0 (0)	0 (0)	0 (0)
